# Comparison of two novel methods of measuring the blood velocity in the deep veins of the lower leg using phase contrast MR imaging

**DOI:** 10.1186/1532-429X-11-S1-P40

**Published:** 2009-01-28

**Authors:** Iain Pierce, Peter D Gatehouse, Xiao Yun Xu, Jenny Keegan, Andrew D Scott, David N Firmin

**Affiliations:** Imperial College London, London, UK

**Keywords:** Wall Shear Stress, Wave Input, Velocity Encode, Muscle Pump, Average Velocity Profile

## Aim

To compare the effectiveness of two novel methods developed to measure the velocity of the blood in the deep veins in the lower leg.

## Background

To investigate the causes and treatment of deep vein thrombosis, there is a need for blood flow measurements in the lower limb veins. The work of Downie et al into the effects of compression stockings on geometry [1] and wall shear stress [2] concludes that MR imaging should be utilised for continued research of the haemodynamics of the lower limb venous system as it can supply structural and velocity data from the same acquisition. Venous velocity is low and more dependent on the respiratory than cardiac cycle, requiring new methods. Also the muscle pump can cause large spikes in the velocity requiring real-time measurement.

## Methods

Two sequences have been developed using Phase-Contrast velocity mapping: gated SEGmented GRadient Echo (SEG-GRE) sequence and an Interleaved SPiral (ISPLASH) sequence. Both use VElocity ENCoding (VENC) of 10 cm/s, arterial flow saturation upstream, slice thickness of 7 mm and pixel size of 1 × 1 mm.

Seven normal volunteers were asked to lie prone (1.5 T Siemens Avanto) with left leg raised 10 – 15 cm, resting the ankle on a foam support, with a pair of carotid surface coils (Machnet) placed around the calf. The volunteers were asked to breathe deeply, in time with an LED driven by a 7 second/cycle square wave input [3]. The logfiles for the subject's respiratory belt sensor were recorded for each spiral scan.

### SEG-GRE

triggered by the square wave input with 7 seconds cine acquisition window (23 respiratory-cine frames) covering one respiratory cycle, acquiring 11 rawdata lines per cine frame per cycle. The velocity encoding gradients are applied every other excitation (REF – VENC) within each cine frame. The number of averages was 2, acquisition time 2.5 minutes, time per frame 285 ms.

### ISPLASH

The ISPLASH sequence uses water excitation and balanced velocity encoding using alternate excitations (VENC (+ve) – VENC (-ve)). It was run with 4 interleaves repeated continuously covering 35 seconds. The spiral data was re-gridded and by sliding reconstruction (In-house Matlab programs) allowed interpolation between repetitions. Time per frame is 320 ms, with interpolation 80 ms between frames.

**Figure 1:**
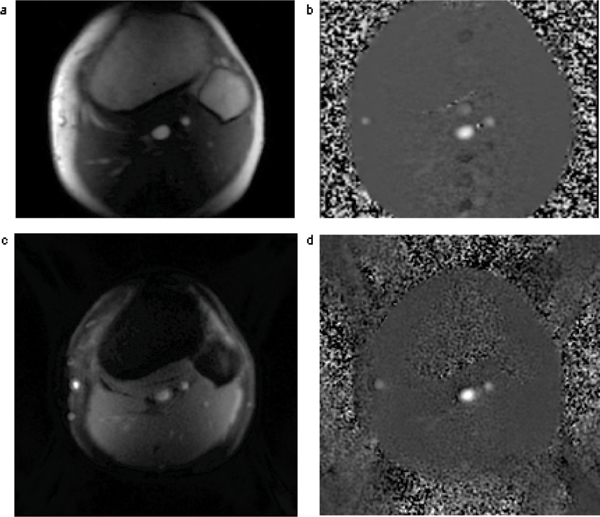
**SEG-GRE magnitude image at peak mean velocity (a) and corresponding PC image (b)**. ISPLASH magnitude image at peak mean velocity (c) and corresponding PC image.

The deep vessels were manually segmented (CMR Tools) from the magnitude images and the regions of interest transferred to the phase images for a mean velocity measurement. For comparison, an average from 4 cycles was taken from the ISPLASH velocities. Figure 1.

## Results and discussion

**Figure 2:**
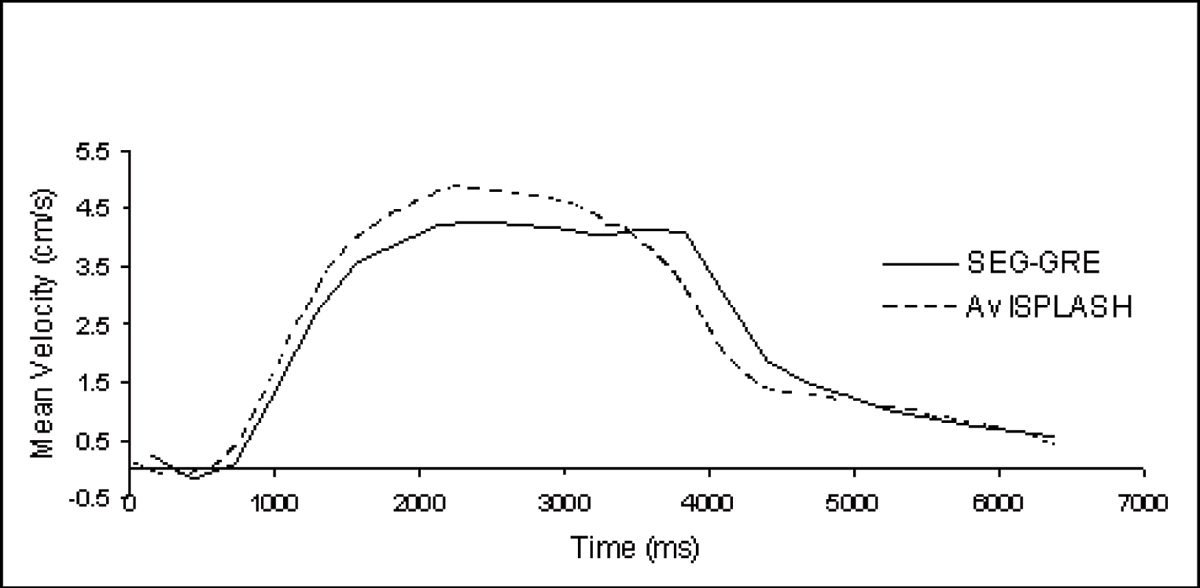
**Mean velocity measured from SEG-GRE and the averaged mean velocity, 4 respiratory cycles, from ISPLASH**.

**Figure 3:**
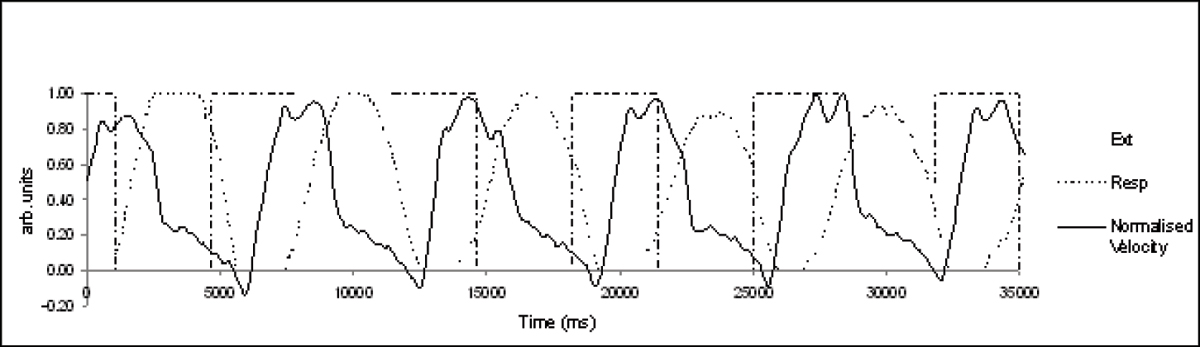
**Full 'real-time' plot of normalised mean velocty with the respiratory (Resp) and the LED square wave used for breathing regulation (Ext)**.

The SEG-GRE and 4-cycle averaged ISPLASH velocity waveforms (Fig. 2) show that both methods yield similar waveforms, similar to those previously seen using Doppler [2, 3]. The peak velocities were taken for each subject from the SEG-GRE, the averaged and real-time ISPLASH waveforms (Table 1). While the average values agree closely, the results also show that the ISPLASH method reveals faster peaks of venous flow. These are averaged down in the SEG-GRE scan due to the number of cycles per image and can be duplicated with 30 sec of ISPLASH data. Also the 'real-time' data (Fig. 3) probably shows cardiac pulsations on top of the respiratory based cycle.

**Table 1:** The peak mean velocity measured from SEG-GRE averaged ISPLASH (4 respiratory cycles) and the full ISPLASH

	Peak mean velocity (cm/s)		
Subject	SEG-GRE	Av ISPLASH	ISPLASH
1	2.06	2.39	2.89
2	5.94	6.66	8.64
3	4.94	5.88	6.81
4	8.17	8.15	10.37
5	6.43	7.24	8.98
6	4.3	4.93	5.26
7	6.79	6.63	7.09

## Conclusion

Two methods for measuring the venous blood velocities in the lower leg have been compared and show good agreement. The first method produces an averaged velocity profile over the respiratory cycle but relies on sustained regular breathing. The second method, ISPLASH, allows for more 'real time' imaging allowing further investigation into areas such as the muscle pump and graduated compression devices.
